# Indents

**Published:** 1905-12-15

**Authors:** 


### INDENTS.

**☞ Brief Articles of Technical or Scientific Value will receive notice and comment. Contributions invited.**

**To Groove Gold Inlay, to Attach Cement.** Use a fine pointed plugger point in an engine mallet or electric mallet run at high speed. Change the angles to get contrary spurs to afford better attachment for the cement. —Dr. G. S. Hershey, in *Review*.

**Cover for Bracket Table.** Cut a ground glass to fit nicely within the moulding on top of the table. First cover the top with white paper and lay the glass on it with ground side up. This makes a pleasing and clean table top, which will not show the scratches of instruments.—W. H. Reaben, in *Review*.

**Blind Abscess.** I have had two very stubborn cases of alveolar abscess which I cured with two applications of cresylone. A new cresol preparation made by Parke, Davis & Co. It is a good detergent disinfectant and makes a good root canal application as its effects are not quickly dissipated.—Thomas Sheridan, Jacksonville, Florida.

**Cautions Against the Use of Adrenalin.** M. I. Wilbert, in *American Journal of Pharmacy*. Neugebauer reports that he has seen several cases of localized gangrene following the use of solutions to which adrenalin had been added for the infiltration method of local anesthesia. Elderly persons were especially liable to this, and he therefore cautions against the use of adrenalin in old persons.

**Cocain Solutions.** The toxic effects of cocain depend not only on the quantity of the alkaloid injected, but likewise, and to a great extent, upon the strength of the solution. Reclus, starting with a 20 percent solution, has gradually decreased the percentage until at the present time he employs solutions of from one-half to one percent, the maximum, with the results all that could be derired.—J. E., *Dental Cosmos.*

**Manipulation of Plaster in Vulcanite Work.** Use good plaster in model and instrument, and mix carefully to get density. Provide ample space for excess rubber. Pack carefully and avoid excess of vulcanite. Close flask slowly under dry heat. Place flask above water in vulcanizer, carry heat slowly from 240° to 320°. In this way the plates will be well vulcanized and there will be the slightest change in the form of the model.

**Removal of Vulcanite from Porcelain Teeth.** Cover the bottom of an iron ladle, such as used for melting lead or zinc, with a layer of dry plaster of Paris; lay the teeth to be cleaned on the surface and sprinkle dry plaster over, completely covering them; place over a big bunsen flame in draught chamber or in coke furnace and heat to redness. Then cool down gradually, when on removal the teeth should be found perfectly clean.—Dr. W. T. Findlayson, *Record.*

**Gold and Platinum Alloy.** Take a small piece of 22-karat gold and fuse it with blowpipe and then add small pieces of platinum until it has taken all that is required. This will produce a button of white alloy which is quite brittle. Add of this alloy in required proportion to gold and get any temper of gold desired. Gold clasps as stiff as steel springs may be made in this way that can be rolled or worked to any desired form.—M. G. McElhinney, in *Review.*

**Treatment of Pyorrhea Alveolaris.** The author reports favorably on the treatment of this malady. His method consists in cauterizing the diseased sockets with the thermo-cautery and in injecting between the roots and alveoli solutions of adequate strength of mercury bichlorid in distilled water. This procedure, together with the firm splinting of the teeth, is considered by Mons. Barrie as a method *par excellence* in the therapeutics of pyorrhea alveolaris.—*Cosmos.*

**To keep a Gingival Margin Cavity Dry without Rubber Dam.** R. E. Sparks, in *Review.* Prepare the cavity and place a napkin in position. Saturate a little floss silk, or small, loosely-twisted thread of absorbent cotton with thin cement. Dry the cavity and pack the silk or cotton around under the gum margin. This method is often useful where the rubber and clamp are in position and the rubber is stretched and does not pass under the gum margin at the sides of the clamp.

**Protection of Wounds with Zinc Chlorid.** Brose's researches were conducted on rabbits and demonstrated convincingly that a wound cauterized with a fifty percent solution of zinc chlorid is protected against infection. Even with extremely virulent infection, and when an interval of a minute had elapsed between the wound and the cauterizing, the animals were absolutely protected. Notwithstanding this his researches failed to show that zinc chlorid can be regarded as a disinfectant, even in the strongest solutions. His researches further demonstrated that the results of infection differ accordingly as the germs come in light contact with the wound or are driven forcibly into it.

**Dishonest Practices.** One of the most flagrant cases of actual dishonesty that ever came to my notice happened recently. A lady went to a so-called dentist—he had a diploma from a recognized dental college—and after about one hour of painless operating he informed her that he had inserted seven fillings. She paid her bill and thanked him profusely for being so gentle. Upon examining her mouth a few months later no trace of more than eight fillings could be found, all of which were located either in "pin-head" cavities made with one cut of a bur or in natural fissures from which the original caries had not been removed. —F. R. Henshaw, in *Digest*.

**Difficulties in Sterilizing Dental Instruments.** Two reasons have operated against the universal sterilization of dental instruments. First, the instruments themselves, and second, the knowledge that while our instruments may be sterile, the field of operation is almost an incubator of bacteria. An aseptic operation, in the true sense of the word, is practically impossible in dentistry. But, realizing that any break or incision of tissue that we may become infected with bacteria around the site of the wound, we must be positive that we do not produce any. In other words, while we cannot insure our patients *against* infection, we can, and must insure that we do not *supply the infection*.—Dr. S. H. Voyles, *Brief*.

**Tin.** When I was a student in Philadelphia in 1871-2, tin was largely used for filling teeth, and every operator of experience knows that a good tin filling in certain locations will preserve a tooth from further decay, or, rather, arrest the progress of caries, as well as any material known. Every student was required to make twenty-five tin fillings. Not an amalgam filling was inserted during my college course in the Pennsylvania College of Dental Surgery. This was at a time when amalgam was very unpopular. The college faculty did not teach its use. Tin has been revived to some extent in the past decade in combination with gold for cervical borders, but it is a matter of regret that tin is not used in many places where amalgam is now employed. I regard it unquestionably superior to amalgam as a preservative of tooth structure. —*Review.*

**The Best Course of Study for a Dentist.** When Dr. James said that the best course of dental study is the longest course of study that will continuously improve the average candidate for the practice of dentistry, regarded from the standpoint of primary and professional education and technical skill, he gave us something to think about. It is worth studying well and with care. Of course, there are great differences in men, but when he said the average candidate, he limited it properly. We cannot set up a scheme that will be best adapted to the brightest men and try to force all others up to it, and yet we must not allow the average man to be of too low a grade. His primary education ought to be up to a certain standard, he ought to show certain aptness in educational and technical matters, and our average man ought to be a good man; a strong man in most directions.—G. V. Black.

**Amalgam.** Amalgam has been improved very much in the last fifteen years. The study of expansion and contraction, the resisting force and so forth, enable us to better understand its behavior, and how to use it more intelligently than formerly. Notwithstanding the improvement in the quality of amalgams, which, no doubt, has been a great benefit to dentistry, the wide publicity of experiments in testing amalgams, in my opinion, has increased and extended its use to an extent that is unjustifiable. Amalgam is not a good material with which to fill teeth. Even the most improved amalgam is unreliable. While it will sometimes remain in a cavity for many years, and the tooth not break down or decay, such cases are not numerous. Nearly all cavities filled with amalgam have recurring decay.—Dr. Brophy.

**New Property of Aluminium.** "A German Investigator," says the *Scientific American*, "has discovered an exceedingly valuable and important property of aluminium, which consists in its application as a whetting agent, the effect produced on cutlery set with it being most astonishing. Though a metal, aluminium possesses the structure of a fine stone, has a strong dissolving power, and develops, upon use for honing, an exceedingly fine metal-setting substance of greasy feel, while showing great adhesion to steel. The knives, etc., treated with it quickly obtain such a fine, razor-like edge, that even the best whetstone cannot produce a like result. Thus, knives which had been carefully set on a whetstone, when magnified a thousand times, still exhibit irregularities and roughness on the edge, while the edge of knives sharpened on aluminium, upon exactly the same magnification, appeared as a straight, smooth line."—*Digest.*

**Prophylaxis of Mercurial Stomatitis.** The prevention of that type of stomatitis brought about indirectly by the elimination of mercury through the mucous membrane of the mouth is the subject of Dr. Almkvist's interesting communication. The preventive treatment consists in placing the teeth in as healthy a condition as possible before the mercurial treatment is begun. The gums should be massaged daily, using alcohol in connection with the friction movements, for, as is well known, this agent has the power of hardening tissues. The teeth should be kept scrupulously clean and brushed frequently with the following paste:

| | | |
|---|---|---|
| Potassium chlorate | gm. | xxxvi. |
| Sodium benzoate | " | iii. |
| Powdered white soap | " | iv. |
| Sodium biborate, Glycerin, | aa " | viij. |
| Aromatic essences | " | j. |

**Infected Instruments.** Is there a set of instruments used solely in dental operations which is especially likely to transmit infection? There is. I believe the danger of transmitting such disease as syphilis by the dentist has been much overrated, while the most common dental infection by which I mean infection caused by the dentist, has been too little studied. The instrument, in the barbs of which lurks the secret foe, is the canal broach. I have seen an ordinary, bright steel needle dipped in pus, wiped on a napkin, stuck once in sterilized gelation, and within a brief period a fine colony of germs has been seen to grow. Bear this in mind and think of the barbed broach, used in some foul canal, and later employed to remove a pulp from a healthy tooth. Is it not a self-evident proposition that if this is done, and it is a grave question whether it should be, it is absolutely the dentist's duty to thoroughly sterilize that broach between the two operations? Is it done?—Dr. Ottalenqui, in *Digest.*

**What the Prophylaxis Treatment is.** Let us here consider the nature and character of the prophylaxis treatment, and what it does for the mouth and teeth. In general the treatment consists of enforced, radical and frequent change of environment for all teeth and all mouth conditions, and the maintenance of perfect sanitation for the oral cavity. More in detail it is the careful and complete removal of all concretions, calcic deposit, semisolids, bacterial plaques and inspissated secretions and excretions which gather on the surfaces of the teeth, between them, or at the gum margin; this instrumentation to be followed in every case by the thorough polishing of all tooth surfaces by hand methods—power polishers should never be used—not alone the more exposed labial and buccal surfaces of the teeth but the lingual, palatal and proximal surfaces as well, using for this purpose orange wood points in suitable holders—porte-polishers—charged with finely ground pumice stone as a polishing material. Treated in this manner the teeth are placed in the most favorable condition to prevent and repel septic accumulations and deposits, and what is of equal importance, it aids the patient in all efforts to maintain sanitation and cleanliness.—Dr. D. D. Smith, Extract, *Briefs.*

**Accidents.** July 11th, a man at Petersburg, Ind., swallowed a plate carrying three false teeth and for some time was in a serious condition.—July 25, a gasoline tank exploded in the office of a dentist at Clinton, Iowa, setting fire to the woodwork, but little damage was done.—Aug. 4th, a vulcanizer exploded in the office of J. H. Dwight, at Des Moines, Iowa, causing a great deal of damage. Dr. Dwight had just stepped out of the laboratory when the explosion occurred, so was not injured.—A. N. Martin, a dentist at Lebanon, Mo., recently broke the skin of his hand on the sharp corner of a patient's decayed tooth. Soon after blood-poisoning set in and he had to visit a specialist at St. Louis, but is now reported to be almost well.—July 1, a man at Tonawanda, N. Y., swallowed his false teeth while eating. His plate was cracked and he had been warned not to use it, but he thought he would save the dentist's fee as long as he could, so continued to use it. The latest reports are that half the plate has been located in the throat and the other half in the stomach, so that two operations may be necessary. —July 25, a man at Springfield, O., swallowed his false teeth while eating and an operation was necessary to relieve him. —July 22, a traveling dentist, who advertised to "extract teeth by a new and secret painless method," pulled twenty-three teeth for a boy nineteen years old. A few hours later the young man was in terrible agony from lacerated jaws, inflammation and an overdose of cocain, and two physicians were in constant attendance on him for twenty-four hours. The "painless extractor" left town suddenly on hearing of the matter.—*Digest.*

